# Virus-Like Particle Facilitated Deposition of Hydroxyapatite Bone Mineral on Nanocellulose after Exposure to Phosphate and Calcium Precursors

**DOI:** 10.3390/ijms20081814

**Published:** 2019-04-12

**Authors:** Olga V. Sinitsyna, Valentine V. Makarov, Kara McGeachy, Tatyana Bukharova, Eric Whale, David Hepworth, Igor V. Yaminsky, Natalia O. Kalinina, Michael E. Taliansky, Andrew J. Love

**Affiliations:** 1 A.N. Nesmeyanov Institute of Organoelement Compounds of Russian Academy of Sciences, Moscow 119991, Russia; sinitsyna@gmail.com; 2 A.N. Belozersky Institute of Physico-Chemical Biology, Lomonosov Moscow State University, Moscow 119991, Russia; makarovvalentine@gmail.com (V.V.M.); kalinina@genebee.msu.su (N.O.K.); 3 Shemyakin-Ovchinnikov Institute of Bioorganic Chemistry, Moscow 119991, Russia; Michael.Taliansky@hutton.ac.uk; 4 Cell and Molecular Sciences, The James Hutton Institute, Dundee DD2 5DA, UK; Kara.McGeachy@hutton.ac.uk (K.M.); Tatyana.Bukharova@hutton.ac.uk (T.B.); 5 CelluComp Ltd., Unit 3, West Dock, Harbour Place, Burntisland KY3 9DW, UK; eric.whale@cellucomp.com (E.W.); david.hepworth@cellucomp.com (D.H.); 6 Physical Faculty, Lomonosov Moscow State University, Moscow 119991, Russia; i.yaminsky@gmail.com

**Keywords:** plant virus, structure, biotechnology, mineralization, composite materials

## Abstract

We produced and isolated tobacco mosaic virus-like particles (TMV VLPs) from bacteria, which are devoid of infectious genomes, and found that they have a net negative charge and can bind calcium ions. Moreover, we showed that the TMV VLPs could associate strongly with nanocellulose slurry after a simple mixing step. We sequentially exposed nanocellulose alone or slurries mixed with the TMV VLPs to calcium and phosphate salts and utilized physicochemical approaches to demonstrate that bone mineral (hydroxyapatite) was deposited only in nanocellulose mixed with the TMV VLPs. The TMV VLPs confer mineralization properties to the nanocellulose for the generation of new composite materials.

## 1. Introduction

There is much demand for natural renewable biomass derived nanomaterials not only due to their revalorization of waste streams, but also their broad range of customizable physical and chemical properties which can be deployed to different market sectors. One such nanomaterial are nanocelluloses which can be derived from celluloses of waste herbaceous plants, wood and bacteria, using a plethora of different chemical, mechanical, and enzymatic disruption methods. They are typically inexpensive to produce from waste materials, have low toxicity, biodegradability and biocompatibility, and are thus safe and environmentally friendly materials [1]. These nano dimensional cellulosic structures maybe produced as nanocrystals, nanoplatelets, and nanofibrils, for example, and these are typically high strength materials with large surface areas which are amenable to chemical modification. These aspects can be flexibly tuned during manufacture to produce formulations of nanocellulose that exist as solid blocks, foams, suspensions, printed substrates, sheets and woven fibres. At an industrial level, such formulations have been used as rheology modifiers (as in paints and cements), stabilizing agents, and as durable and high strength materials and composites and also in food applications [2,3].

From the perspective of medicine and biosciences the tunable chemistries of nanocelluloses have been exploited to incorporate reactive biologics that may be utilized as new nanomaterials for controlled drug release, wound repair, tissue engineering, enzymatic bioreactor formulations, and antimicrobial components. Incorporation of biologics into nanocellulose in some situations may be a simple mixing process which could just rely on factors such as pH, surface charge, and hydrophobicity to facilitate attachment. For example, lysozyme mixed with cationic nanocellulose fibres had increased retention, stability, and enhanced antibacterial activities when compared with lysozyme mixed with unmodified or anionic nanocelluloses [4]. Integration into nanocellulose may also be regulated by appropriate protein folding and surface display of hydrophobic groups and their subsequent interaction with hydrophobic regions of nanocellulose, as was suggested by the differential incorporation of elastin-cartilage coiled coil (EC) or cartilage coiled coil-elastin (CE) assembling domains [5]. Where such interactions between the proteins and nanocellulose are quite weak, attachment may be improved using various chemical-linker approaches [6,7,8,9].

In addition to incorporating proteins into the nanocellulose matrices, there has been a wide interest in recent years in coating these structures with bone minerals such as hydroxyapatite (HA). These nanocellulose-HA materials have potential bone repair applications [10,11] and constitute novel nanomaterials and coating which may provide corrosion [12] and water resistance [13], for example. It was previously found that a complex chemical replacement of the hydroxyl groups by phosphate groups in the nanocellulose was required for the deposition of hydroxyapatite (HA) bone mineral (Ca_10_(PO_4_)_6_(OH)_2_), after exposure to precursors such as CaCl_2_ at non supersaturating concentrations. Such deposition was not observed with unmodified nanocelluloses which indicates that the hydroxyl groups are not reactive enough to grow HA crystals [14,15]. It was suggested that the introduced anionic phosphates can interact with the Ca^2+^ ions in the salt, producing nuclei for seeding HA crystal growth [15].

In this paper we describe a novel approach for the facilitated deposition of HA onto plant nanocelluloses, a substrate which would not typically support HA nucleation, by incorporating virus-like particles (VLPs) derived from the Tobacco mosaic virus (TMV) coat protein subunit (CP). TMV VLPs can be easily generated by expressing in bacteria a modified CP (E50Q and D77N mutations) which can assemble into 18 nm diameter, 100–1000 nm length rods in the absence of viral RNA [16]. These particles have the advantage in that they can rapidly be produced to high yields, are non infectious and can be suitably “decorated” with different activities by fusing peptides and proteins of interest to various regions of the coat protein [16]. In this work we found that “non decorated” TMV VLPs had a negative surface charge, which is consistent with previous work on wildtype TMV [17,18] and that they could bind Ca^2+^ ions. We observed that these VLPs could robustly integrate into nanocellulose matrices via a simple mixing step and that they could confer HA mineralizing properties to the nanocellulose upon exposure to calcium and phosphate salts. Chemical analysis confirmed that the crystalline structures on the nanocellulose/VLPs were HA. Thus non infectious virus derived structures can be utilized to alter the properties of nanocellulose to facilitate bone mineral deposition for formation of new composites.

## 2. Results

### 2.1. Production and Isolation of Tobacco Mosaic Virus Derived Virus-Like Particles (TMV VLPs)

**Figure 1:**
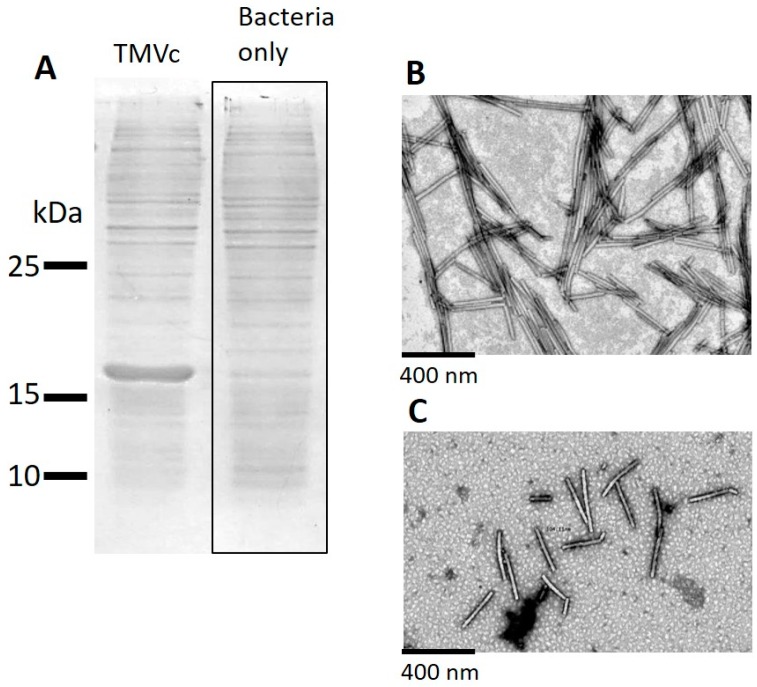
(**A**) Coomassie stained SDS PAGE of lysates derived from BL21(DES)pLysS bacteria only or those expressing tobacco mosaic virus derived virus-like particles (TMV VLPs) (TMVc); molecular weights are indicated on the left side. (**B**) Transmission electron microscopic analysis of TMV VLPs, and (**C**) wild type TMV particles, with scale bars shown.

Bacteria which expressed the TMV CP subunit with the modified E50Q and D77N [16], which is purported to self-assemble into TMV VLPs, were lysed and the lysates were run on sodium dodecyl sulphate polyacrylamide gel electrophoresis (SDS PAGE) gels that were subsequently Coomassie stained (Figure 1A). In the TMV VLP lysates a band was visible at the expected ~17 kDa. No bands of this size were visible in the uninduced bacterial lysate sample. Following successful expression, we subsequently purified the particles from the lysates according to the materials and methods. Transmission electron microscopic (TEM) analysis confirmed that TMV VLPs were successfully isolated as indicated by the presence of 18 nm diameter rods of variable length (Figure 1B); which is in contrast to wildtype TMV which is of 18 nm diameter and has a fixed length of 300 nm (Figure 1C). These findings are broadly consistent with the bacterially expressed modified E50Q and D77N TMV CP produced by Brown et al. [16]. This data was also confirmed via Atomic Force Microscopy (AFM), as seen in Figure S1.

### 2.2. Analysis of the TMV VLP Surface Charges and Association with Calcium

**Figure 2:**
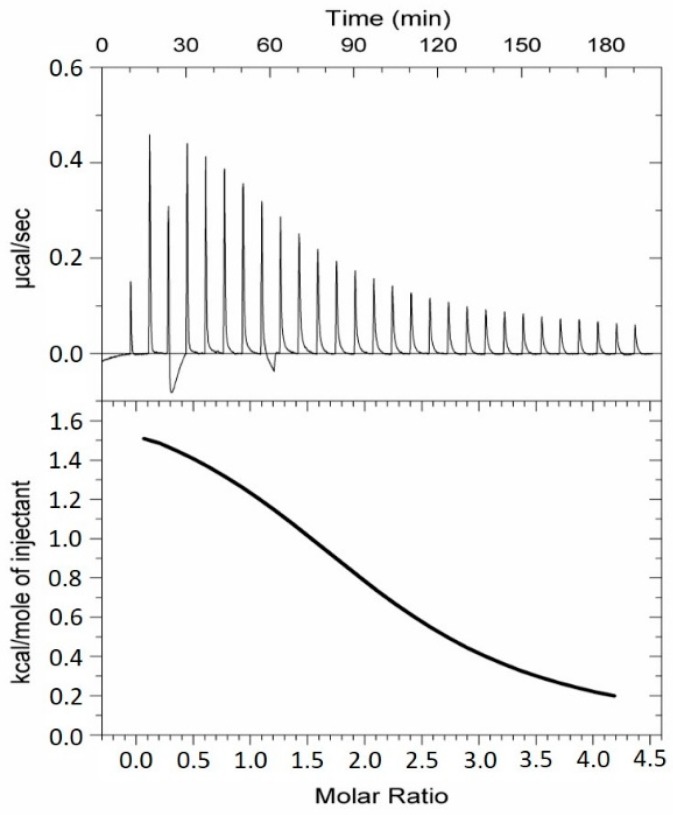
Isothermal titration calorimetry curves of the interaction between CaCl_2_ and TMV VLPs. 0.05 mM TMV VLPs in water were present in the assay vessel and 1 mM CaCl_2_ was used as the injectant. Data was acquired over a period of 3 h.

It has previously been reported that negative surface charges can promote binding of Ca^2+^ and subsequent nucleation of Ca^2+^ and phosphates to form HA [19]. We measured the zeta potential of the TMV VLPs and found that it had a net negative charge of −12.5 mV, which is indicative that it may associate with Ca^2+^; this was subsequently assessed using isothermal titration calorimetry (ITC). The ITC binding curves were produced by monitoring temperature changes in a reaction vessel containing 0.05 mM TMV VLPs in water, to which 1 mM CaCl_2_ was periodically added over time (Figure 2). From this data it was found that the TMV VLPs can bind two calcium ions per subunit in solution, and that this strength of binding (*K*a = (0.25 ± 0.05) × 10^4^ M^−1^) is reminiscent to that observed in moderately strong enzyme-substrate and enzyme-inhibitor interactions.

### 2.3. Analysis of the Attachment of TMV VLPs to Nanocellulose

**Figure 3:**
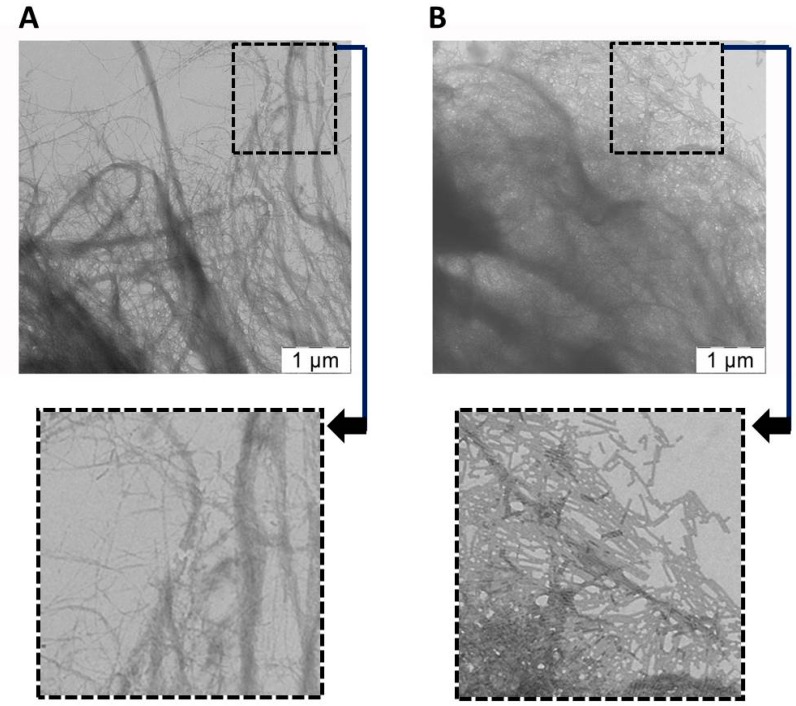
Transmission electron microscopic analysis of (**A**) nanocellulose only or (**B**) TMV VLPs mixed with nanocellulose and washed. Zoomed in regions are indicated by dotted boxes and arrows. Scale bars are provided.

We were interested if this Ca^2+^ binding functionality could be introduced into nanocellulose, in order to produce a new material which has potential mineralization properties. For this process it is crucial that the VLPs are able to closely interact with the nanocellulose, and this was tested initially by mixing the TMV VLPs with the nanocellulose, washing three times, and then examining the association under TEM. It was found that the unmixed nanocellulose samples comprised long micron-sized filamentous strands of diverse thickness (Figure 3A). In contrast, those samples containing TMV VLPs (Figure 3B) had their filamentous structures partially masked by the TMV VLPs, which is suggestive of interaction between the VLPs and nanocellulose. As an additional analysis the samples were dried down for AFM and it was found that the nanocellulose formed a densely packed matrix with discernable fibres (see Figure S2), whereas those containing the TMV VLPs had a surface largely occupied by the TMV VLPs with some visible nanocellulose fibres (see Figure S3).

**Figure 4:**
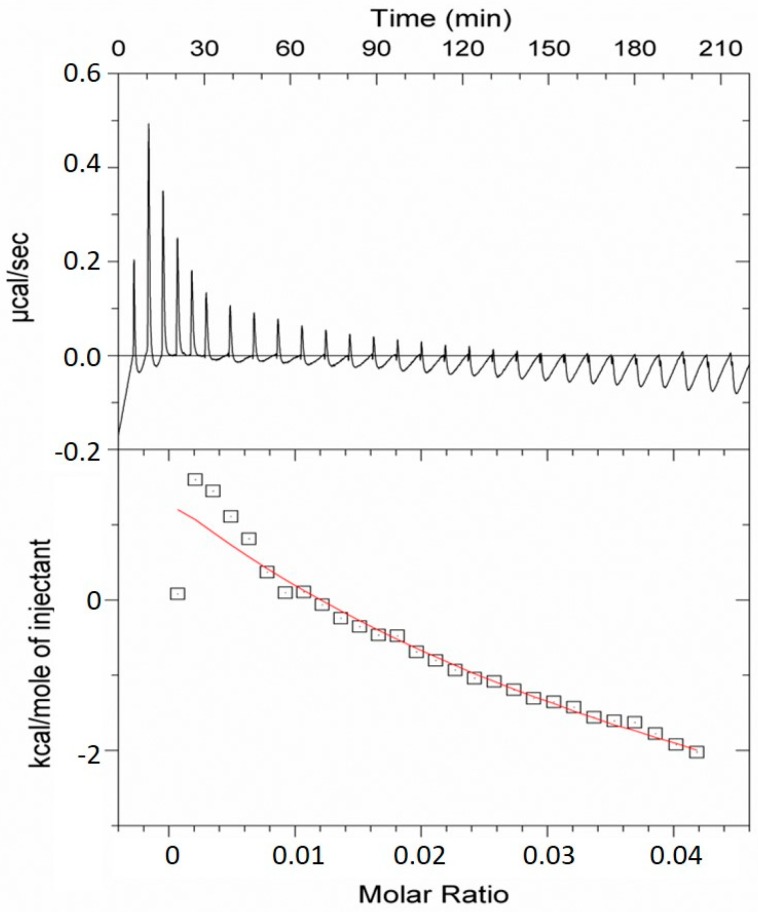
Isothermal titration calorimetry curves of interaction between nanocellulose and TMV VLPs over time. 10 mg/mL of nanocellulose was present in the assay vessel and 5 mg/mL TMV VLPs were used as the titrant. Data was collected over a 3.5 h period.

To more robustly characterize these interactions ITC was performed on nanocellulose mixed with the VLPs (Figure 4; see materials and methods section for more details), and it was found that 10 mg of nanocellulose could bind 0.8 mg of TMV VLPs in a moderately strong manner (*K*a = (0.20 ± 0.02) × 10^4^ M^−1^). *In toto* this demonstrates a strong association and integration of the VLPs into the nanocellulose matrix.

### 2.4. Assessment of Hydroxyapatite Formation on Nanocellulose and Nanocellulose-TMV VLP Composites

**Figure 5:**
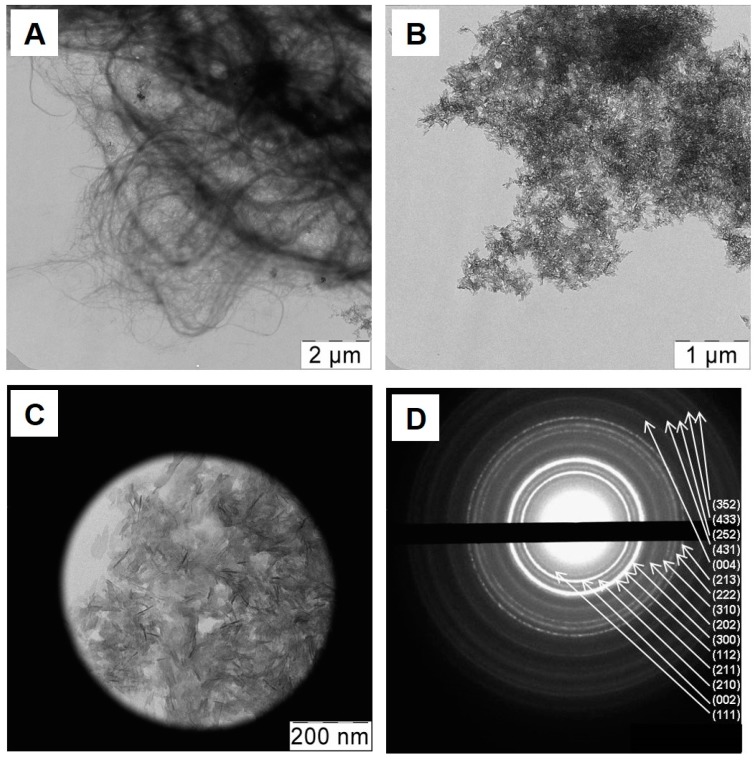
Transmission electron microscopic analysis of (**A**) nanocellulose after exposure to hydroxyapatite precursor salts (100 mM CaCl_2_ pH 4.83 and 60 mM Na_2_HPO_4_ pH 8.36), and (**B**) nanocellulose mixed with TMV VLPs, after exposure to hydroxyapatite precursor salts (100 mM CaCl_2_ pH 4.83 and 60 mM Na_2_HPO_4_ pH 8.36). Picture (**C**) shows selected area in (**B**) from which electron diffraction (**D**) was obtained.

To test that the TMV VLPs can promote HA formation on the nanocellulose networks, hydroxyapatite deposition assays were performed on the matrices by exposing them to 100 mM CaCl_2_ and 60 mM Na_2_HPO_4_ precursors. These were then analysed using TEM, selected area electron diffraction (SAED) and X-ray diffraction (XRD) approaches. Under TEM it was found that the nanocellulose only sample exposed to the HA precursors had the characteristic filamentous structure (Figure 5A) which was consistent to that observed in previous experiments (see Figure 3). There was no obvious crystal formation in this sample; thus indicating that nanocellulose only is incapable of supporting HA deposition from precursors. In contrast, TEM analysis of the nanocellulose into which the TMV VLPs was integrated, had significant crystal deposition which masked the nanocellulose material (Figure 5B); this was consistent with the AFM studies performed on the nanocellulose/TMV VLPs material onto which HA had been deposited (see Figure S4). SAED analysis of this material revealed diffraction rings consistent with the hydroxyapatite crystalline phase arrangement (Figure 5C,D). From the AFM and TEM data we estimated that the crystals were plate-like with diameters ranging between 10–40 nm and a thickness of 2–4 nm.

**Figure 6:**
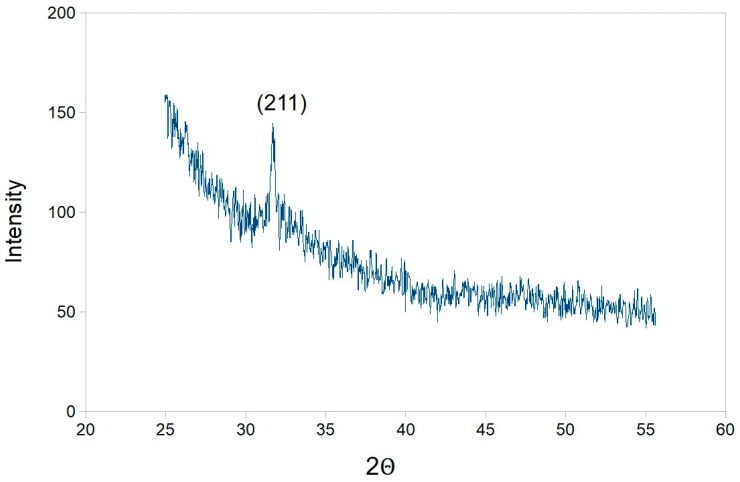
Powder X-ray diffraction on nanocelluloses that contain TMV VLP after mineralization with hydroxyapatite precursor salts. Data were collected over a 2θ range from 20° to 60°, with the characteristic 211 peak for hydroxyapatite indicated (JCPDS No. 9–0432).

The XRD characterization (Figure 6) of these mineralized matrices produced spectra with a peak around 31.7°, which corresponds to (211) planes of the hydroxyapatite crystalline structure (JCPDS No. 9-0432). No other crystalline compounds were detected in the samples. Thus the TMV VLPs, after incorporation into nanocellulose can induce hydroxyapatite mineralization after exposure to salt precursors. Application of the Scherrer equation (*D*_hkl_ *=* 0.9λ/*B*cosθ_hkl_) on the XRD data was used to extrapolate the sizes of the hydroxyapatite crystals and it was found the TMV VLPs/nanocellulose had average HA crystal grain sizes of 8 nm, which is of a similar magnitude to the measurements obtained using microscopy. Thus incorporation of non-infectious TMV VLPs into the nanocellulose network promotes its hydroxyapatite mineralization after exposure to precursor salts.

## 3. Discussion

### 3.1. Surface Chemistry and Mechanisms of Calcium Binding Activities of the TMV VLPs

We measured the zeta potential of the TMV VLPs and found that it had a net negative charge of −12.5 mV, a result which is generally consistent with earlier findings which report that the wildtype TMV virus is negatively charged at neutral pH levels [20]. Previous work has indicated that the zeta potential of TMV becomes more positive at circumneutral pH as the ionic strength of the solution increases [20]; a phenomenon suggested to be due to its association with cations in the buffer. Indeed experimental data from prior work [17,18] has demonstrated that apart from the two internal metal binding sites present in the TMV CP subunit, the assembled virus is decorated with surface exposed cation binding sites (which may sequester Ca^2+^), comprising C-terminal carboxyls, aspartic acid and glutamic acid residues (which are found at amino acid positions 64 and 145 respectively in the TMV CP subunit; a cartoon of this schematic is shown in Figure S5). Interestingly these Ca^2+^ sequestering amino acids are also found in preponderance in various classes of protein such as osteopontin, dentin matrix protein 1 and bone sialoprotein, which have been implicated in hydroxyapatite deposition. Such proteins are thought to promote hydroxyapatite mineralization through initial association with Ca^2+^ and then PO_4_^3−^ ions [19,21]. However there are many other proteins which have long charged regions of these amino acids but they are quite ineffectual at promoting HA deposition; indicating that the protein conformation in addition to the amino acid sequence regulate HA formation [19,21,22,23]. In the case of the TMV VLPs, we would suggest that the close proximity of C-terminal carboxyl, aspartic acid (position 64) and glutamic acid (position 145) residues to one another on each subunit (see Figure S5) are in a suitable conformation to promote HA deposition. Moreover, given that 2130 coat protein subunits constitute a 300 nm length VLP, and that these amino acids are surface available on each subunit, this indicates that the VLPs could trigger multiple HA nucleation and aggregation events.

Given the importance of association of Ca^2+^ with charged amino acids for hydroxyapatite nucleation, we assessed the level of interaction between TMV VLPs and Ca^2+^ using ITC. The data we obtained indicated that Ca^2+^ can bind the TMV VLPs strongly, likely via the negatively charged aspartic acid, C-terminal carboxyls and glutamic acid residues on the TMV VLP surface. This is consistent with the corpus data [17,18] and is indicative that TMV VLP could facilitate hydroxyapatite nucleation.

Very recent and elegant work by Lauria et al. [24] utilized the 515 × 13.5 nm flexuous Potato virus X (PVX) as a platform for presenting RGD (an arginine-glycine-aspartic acid peptide which promotes cellular binding via attachment to integrin receptors [25]) on the surface of the virus by fusing this motif to the N-terminus of the coat protein. The authors found that the unmodified PVX was quite inefficient in nucleating HA formation from salt precursors, which was in contrast to the RGD peptide functionalized PVX which could promote significant HA deposition (likely mediated via the aspartic acid). This is completely consistent with the role of R, G, and D amino acids in mineralization described above [17,18] and is also complementary to our findings that TMV VLP particles which have surface exposed aspartic acid residues (though not in an RGD sequence) can promote HA deposition.

### 3.2. Potential Mechanisms of Interaction between Nanocellulose and TMV VLPs

Not only do TMV VLPs have capacity for potential HA nucleation and growth but it can also bind to nanocellulose formulations in a strong manner as demonstrated by the ITC (Figure 4) and associated data. At present, it is not completely clear what the mechanism is which facilitates this attachment, but based on the literature, it probably involves a variety of different interactions of varying contribution. Cellulose has an amphiphilic character due to its anisotropic structure [26,27] of hydroxyl groups at the equator of the d-anhydroglucopyranose rings and hydrogen atoms at axial positions; in effect, producing a hydrophilic equator and hydrophobic axial plane. It is possible that a superfluity of hydroxyl groups present on the nanocellulose [28] may participate in hydrogen bonding with carboxylate groups present on the surface of TMV VLPs, particularly at a pH > 3.5 [29]. In addition, at circum-neutral pH the TMV outer surface has a repeating sequence of positive and negative regions punctuated by hydrophobic domains [30], and these hydrophobic patches may interact with corresponding hydrophobic axial planes of the cellulose.

### 3.3. Formation of New Mineralized TMV VLP-Nanocellulose Composites and Their Future Applications

Taken together the TMV VLPs have the appropriate structure and surface chemistry for attachment to nanocellulose and for the binding of cations, which mediates hydroxyapatite formation. We took the TMV VLP and nanocellulose complexes and exposed them to HA precursor salts, which led to the formation of crystal structures on the complex surface which (as indicated by the TEM and SAED analysis; Figure 5) was chemically characterized as being HA (XRD data; Figure 6). In contrast, the nanocellulose formulation not containing the TMV VLP had no HA deposition after precursor treatment. This is consistent with several other studies which show that modifications of nanocellulose or prolonged incubations of nanocellulose with supersaturating solutions of precursors can be required for HA deposition. For example Gonzalez et al. [14] recognized that the primary hydroxyls of the nanocellulose are not reactive enough to grow HA crystals and they modified these using titanium isopropoxide to convert these into three new highly labile and reactive hydroxyl groups which could support HA nucleation from salt components. Some other studies were able to induce HA formation on nanocellulose by incubating with 0.1 M CaCl_2_ for 3 days, and then exposing to a supersaturating concentration of simulated body fluid which was repeatedly replaced over a 7–14 day period in order to maintain supersaturation [15]. In contrast to these studies the approaches described in our paper can be utilized to nucleate and grow HA crystals on nanocellulose in a timely manner (within 24 h), without having to resort to complex chemical approaches or the maintenance of supersaturating salt conditions over prolonged periods. For such applications the TMV VLPs can be bulk produced in bacteria.

Bone engineering has been suggested as a possible application of such materials [10,11], given that the HA is biocompatible and has bone bonding capacity [31] and that the cellulose is also biocompatible and confers strength and resistance to degradation [32]. An interesting note is that the nanocellulose-HA material we produced contains non infectious rods derived from the TMV CP subunit, and it has previously been shown that wildtype TMV particles can promote biocompatibility and trigger cellular differentiation into osteogenic cells, for enhanced bone growth [33]. To speculate, this may suggest that our materials contain modulatory signals which could improve biocompatibility and bone growth. In future experiments we would test if and how the matrices would support osteoprogenitor cell growth and differentiation into osteogenic cells in culture, which would lead to bone deposition. This would establish, to some extent, how biocompatible and stable/degradable the nanocellulose/TMV VLPs/HA composites are and to what degree the cells are able to penetrate into the matrix. The level of cellular penetration and also experimentation measuring the strength, flexibility, and compactibility of the materials in comparison with real bone would inform us what form the material should be in for potential in vivo studies (for example sheets, blocks, or suspensions).

Another application of such materials is corrosion resistant coatings, particularly with regard to metallic implants [12], with the HA predominantly providing this resistance and the cellulose conferring a structurally stable and mechanically robust network for the attachment of the HA. It is possible that our material may also have some use in this area of research, but like with the potential application to bone repair, our material will have to undergo further work to establish the mechanical properties, durability and potential biocompatability before deployment to such uses.

In summary, mixing a non-infectious biosafe TMV VLP into nanocellulose confers a novel functionality to the nanocellulose, which permits spontaneous hydroxyapatite mineral deposition onto the complex after exposure to salt precursors. This work constitutes the first report of such induction of mineralization on surfaces, catalyzed by a virus-like particle.

## 4. Materials and Methods

### 4.1. Sequences and Plasmids

For the TMV VLPs, the TMV E50Q and D77N group modified coat protein sequence (TMV VLPs; [16]) was codon optimized for bacterial expression and cloned into a pET-21a(+) vector at the *Nde*I/*Xho*I restriction sites with a stop codon to prevent fusion to the neighbouring His Tag by Genscript (Piscataway, NJ, USA). Plasmids were transformed into BL21(DES)pLysS chemically competent cells (ThermoFisher Scientific, Paisley, UK) by following the manufacturers recommendations.

### 4.2. Expression in Bacteria and Isolation of VLPs

Bacteria were grown at 37 °C with shaking at 200 rpm in LB media supplemented with 100 µg/mL ampicillin and 34 µg/mL chloramphenicol. IPTG was added to a final concentration of 0.1 mM, once cultures reached an OD_600_ of 0.5. Cultures were then incubated overnight at 20 °C with shaking at 200 rpm. After centrifugation at 4000× *g* for 15 min, the supernatant was removed, and the bacterial cells were lysed by freeze-thawing 3 times using liquid nitrogen and a 37 °C water bath. Crude lysates were mixed with B-PER Complete reagent (ThermoFisher Scientific; 2 mL B-PER complete added per 5 mL of initial culture used) at room temperature for 30 min prior to centrifugation at 10,000× *g* for 20 min. Six parts of this supernatant was mixed with 1 part chloroform for 10 min at 4 °C, and then centrifuged at 10,000× *g* for 10 min at 4 °C. To the upper phase PEG 8000 and NaCl were added to a final concentration of 2% (*w*/*v*) and 1% (*w*/*v*) respectively. This was left overnight at 4 °C, and then centrifuged at 10,000× *g* for 20 min at 4 °C. The VLP pellet was resuspended in 25 mM tris-HCl (pH 7.8), centrifuged at 10,000× *g* for 10 min at 4 °C to clarify and the supernatants were retained. Supernatants were loaded onto a 20% sucrose 25 mM tris-HCl (pH 7.8) cushion, which was then centrifuged at 175,500× *g* in a SW41 rotor at 4 °C for 2 h. Virus pellets were resuspended in 0.01 M Tris-HCl (pH 7.8), centrifuged at 175,500× *g* in a SW41 rotor at 4 °C for 2 h, prior to resuspension in deionized water.

### 4.3. SDS-PAGE Analysis

Crude bacterial lysates were mixed 1:4 parts with 4× Laemmli loading buffer and then boiled for 10 min to denature the proteins. Twenty microliters of each of these samples were loaded onto 15% SDS-PAGE gels, along with 15 μL of PageRuler Plus prestained ladder (ThermoFisher Scientific). Gels were run at 200 V for a 45 min and then stained in Quick Blue Coomassie stain (Triple Red, Bucks, UK) prior to photography.

### 4.4. Nanocellulose Formulation

Nanocellulose formulation 300315-M 0.69% was obtained from CelluComp (Burntisland, UK).

### 4.5. Hydroxyapatite Mineralization Reactions

Two hundred and fifty microliters of nanocellulose (0.69%) was combined with 20 µL of VLPs (20 mg/mL) and this was added to 125 µL of 100 mM CaCl_2_ and then to 125 µL of 60 mM Na_2_HPO_4_. The reactions were allowed to progress overnight prior to analysis.

### 4.6. Transmission Electron Microscopy, Selected Area Diffraction, X-ray Diffraction and Zeta Potential Analysis

Ten microliter volumes of neat, 1:10 and 1:100 dilutions of VLPs, nanocellulose or VLPs with nanocellulose were pipetted on to 200 mesh carbon coated copper grids and incubated for 30 s. Grids were wicked by using Whatman 3MM blotting paper and washed with 20 µL deionized water prior to application of 20 µL of 2% (*w*/*v*) uranyl acetate. After incubation for 1 min, the grids were wicked and left to air dry. VLPs were then observed in a JEOL 1400 transmission electron microscope (JEOL, Tokyo, Japan) at 80 kV.

Hydroxyapatite mineralization samples were placed on formvar coated copper grids and air dried prior to visual and electron diffraction pattern analysis under a LEO912 AB OMEGA transmission electron microscope (Carl Zeiss, Oberkochen, Germany) operating at 100 kV.

Dry powders of the samples were examined by X-ray diffraction in a DRON-3M X-ray diffractometer (Burevestnik, Saint Petersburg, Russia) (Cu *K*α = 1.5418 Å) operating at 30 kV and 20 mA (wavelength). The diffraction patterns were collected over a 2θ range from 20° to 60°. The Scherrer equation (*D*_hkl_ *=* 0.9λ/*B*cosθ_hkl_) was used to estimate crystal sizes.

Zeta potential measurements were obtained on a Zetasizer Nano ZS device (Malvern Instruments Ltd., Malvern, UK) with sample temperatures maintained at 0.1 °C using a Peltier thermostatting system. Zeta potential measurements were performed on 1 mL of 0.15–0.3 mg/mL concentrations of TMV VLPs in folded capillary cells (Malvern Instruments Ltd., Malvern, UK).

### 4.7. Isothermal Titration Calorimetry

ITC assays were conducted with a MicroCal VP-ITC (Malvern). For assessment of interaction of VLPs with Ca^2+^, VLPs (in deionised water) were placed in a cell in the ITC apparatus at a molarity of 0.05 mM (the molarity is calculated per the VLP subunit) and 1 mM CaCl_2_ was used as an injectant. Data was recorded over a 3 h period. VLP and nanocellulose interactions were carried out in a similar manner except that nanocellulose (10 mg/mL) was placed in the cell and 5 mg/mL of VLPs was used as the titrant.

## Supplementary Materials

Supplementary materials can be found at https://www.mdpi.com/1422-0067/20/8/1814/s1.

## Abbreviations

AFM	Atomic Force Microscopy
CP	Coat protein
HA	Hydroxyapatite
IPTG	Isopropyl β-d-1-thiogalactopyranoside
ITC	Isothermal Titration Calorimetry
PVX	Potato virus X
SDS PAGE	Sodium dodecyl sulphate polyacrylamide gel electrophoresis
SAED	Selected Area Electron Diffraction
TEM	Transmission Electron Microscopy
TMV	Tobacco mosaic virus
VLP	Virus-like particle
XRD	X-ray diffraction

## References

[1] Mondal S. (2017). Preparation, properties and applications of nanocellulosic materials. Carbohydr. Polym..

[2] De France K.J., Hoare T., Cranston E.D. (2017). Review of hydrogels and aerogels containing nanocellulose. Chem. Mater..

[3] Tardy B.L., Yokota S., Ago M. (2017). Nanocellulose-surfactant interactions. Curr. Opin. Colloid Interface Sci..

[4] Uddin K.M.A., Orelma H., Mohammadi P., Borghei M., Laine J., Linder M., Rojas O.J. (2017). Retention of lysozyme activity by physical immobilization in nanocellulose aerogels and antibacterial effects. Cellulose.

[5] Haghpanah J.S., Tu R., Da Silva S., Yan D., Mueller S., Weder C., Foster E.J., Sacui I., Gilman J.W., Montclare J.K. (2013). Bionanocomposites: Differential effects of cellulose nanocrystals on protein diblock copolymers. Biomacromolecules.

[6] Orelma H., Filpponen I., Johansson L., Österberg M., Rojas O.J. (2012). Surface functionalized nanofibrillar cellulose (NFC) film as a platform for immunoassays and diagnostics. Biointerphases.

[7] Zhou J., Butchosa N., Jayawardena H.S.N., Park J., Zhou Q., Yan M.D., Ramstrom O. (2015). Synthesis of multifunctional cellulose nanocrystals for lectin recognition and bacterial imaging. Biomacromolecules.

[8] Saito T., Isogai A. (2004). TEMPO-mediated oxidation of native cellulose. The effect of oxidation conditions on chemical and crystal structures of the water-insoluble fractions. Biomacromolecules.

[9] Oshima T., Taguchi S., Ohe K., Baba Y. (2011). Phosphorylated bacterial cellulose for adsorption of proteins. Carbohydr. Polym..

[10] Beladi F., Saber-Samandari S., Saber-Samadari S. (2017). Cellular compatibility of nanocomposite scaffolds based on hydroxyapatite entrapped in cellulose network for bone repair. Mater. Sci. Eng..

[11] Ao C., Niu Y., Zhang X., He X., Zhang W., Lu C. (2017). Fabrication and characterization of electrospun cellulose/nano-hydroxyapatite nanofibers for bone tissue engineering. Int. J. Biol. Macromol..

[12] Zhong Z., Qin J., Ma J. (2015). Cellulose acetate/hydroxyapatite/chitosan coatings for improved corrosion resistance and bioactivity. Mater. Sci. Eng. C.

[13] Ishikawa M., Oaki Y., Tanaka Y., Kakisawa H., Salazar- Alvarez G., Imai H. (2015). Fabrication of nanocellulose-hydroxyapatite composites and their application as water-resistant transparent coatings. J. Mater. Chem. B.

[14] Gonzalez M., Hernandez E., Ascencio J.A., Pacheco F., Pacheco S., Rodriguez R. (2003). Hydroxyapatite crystals grown on a cellulose matrix using titanium alkoxide as a coupling agent. J. Mater. Chem..

[15] Wan Y.Z., Hong L., Jia S.R., Huang Y., Zhu Y., Wang Y.L., Jiang H.J. (2006). Synthesis and characterization of hydroxyapatite-bacterial cellulose nanocomposites. Compos. Sci. Technol..

[16] Brown A.D., Naves L., Wang X., Ghodssi R., Culver J.N. (2013). Carboxylate-directed in vivo assembly of virus-like nanorods and tubes for the display of functional peptides and residues. Biomacromolecules.

[17] Gallagher W.H., Gastfriend H.H., Lauffer M.A. (1983). Ion binding by TMV, TMV protein and TMV-RNA. Biophys. J..

[18] Nedoluzhko A., Douglas T. (2001). Ordered association of tobacco mosaic virus in the presence of divalent metal ions. J. Inorg. Biochem..

[19] Choi Y.S., Lee J.Y., Suh J.S., Lee G., Chung C.P., Park Y.J. (2013). The mineralization inducing peptide derived from dentin sialophosphoprotein for bone regeneration. J. Biomed. Mater. Res. A.

[20] He J., Niu Z., Tangirala R., Wan J.Y., Wei X.Y., Kaur G., Wang Q., Jutz G., Boker A., Lee B. (2009). Self-assembly of tobacco mosaic virus at oil/water interfaces. Langmuir.

[21] Tavafoghi M., Cerruti M. (2016). The role of amino acids in hydroxyapatite mineralization. J. R. Soc. Interface.

[22] Benesch J., Mano J., Reis R. (2008). Proteins and their peptide motifs in acellular apatite mineralization of scaffolds for tissue engineering. Tissue Eng..

[23] Palmer L., Newcombe C., Kaltz S., Spoerke E., Stupp S. (2008). Biomimetic systems for hydroxyapatite mineralization inspired by bone and enamel. Chem. Rev..

[24] Lauria I., Dickmeis C., Röder J., Beckers M., Rütten S., Lin Y.Y., Commandeur U., Fischer H. (2017). Engineered Potato virus X nanoparticles support hydroxyapatite nucleation for improved bone tissue replacement. Acta Biomater..

[25] Ruoslahti E., Pierschbacher M.D. (1986). Arg-Gly-Asp- A versatile cell recognition signal. Cell.

[26] Yamane C., Aoyagi T., Ago M., Sato K., Okajima K., Takahashi T. (2006). Two different surface properties of regenerated cellulose due to structural anisotropy. Polym. J..

[27] Yamane C. (2015). Structure formation of regenerated cellulose from its solution and resultant features of high wettability: A review. Nordic Pulp Pap. Res. J..

[28] Khazraji A.C., Robert S. (2013). Self-assembly and intermolecular forces when cellulose and water interact using molecular modeling. J. Nanomater..

[29] Knez M., Sumser M.P., Bittner A.M., Wege C., Jeske H., Hoffmann D.M.P., Kuhnke K., Kern K. (2004). Binding the tobacco mosaic virus to inorganic surfaces. Langmuir.

[30] Britt D.W., Buijs J., Hlady V. (1998). Tobacco mosaic virus adsorption on self-assembled and Langmuir-Blodget monolayers studied by TIRF and SFM. Thin Solid Films.

[31] Li L., Zhao M., Li J., Zuo Y., Zou Q., Li Y. (2015). Preparation and cell infiltration of lotus-type porous nano-hydroxyapatite/ polyurethane scaffold for bone tissue regeneration. Mater. Lett..

[32] Saber-Samandari S., Saber-Samandari S., Gazi M., Cebeci F.C., Talasaz E. (2013). Synthesis, characterization and application of cellulose based nano-biocomposite hydrogels. J. Macromol. Sci. Pure Appl. Chem..

[33] Sitasuwan P., Lee L.A., Bo P., Davis E.N., Lin Y., Wang Q. (2012). A plant virus substrate induces early upregulation of BMP2 for rapid bone formation. Int. Biol..

