# Supplementary material for: Virus-Like Particle Facilitated Deposition of Hydroxyapatite Bone Mineral on Nanocellulose after Exposure to Phosphate and Calcium Precursors
Source: Int J Mol Sci. 2019 Apr 12;20(8):1814. doi: 10.3390/ijms20081814 (PMC6515374; doi:10.3390/ijms20081814)
Supplement: Supplementary file 1 [file ijms-20-01814-s001.pdf]

Atomic Force Microscopy

Ten microliter volumes of TMV VLPs, nanocellulose or TMV VLPs with nanocellulose were pipetted on to freshly cleaved mica. The samples were dried in air and observed in a FemtoScan atomic force microscope using fpN11 cantilevers.

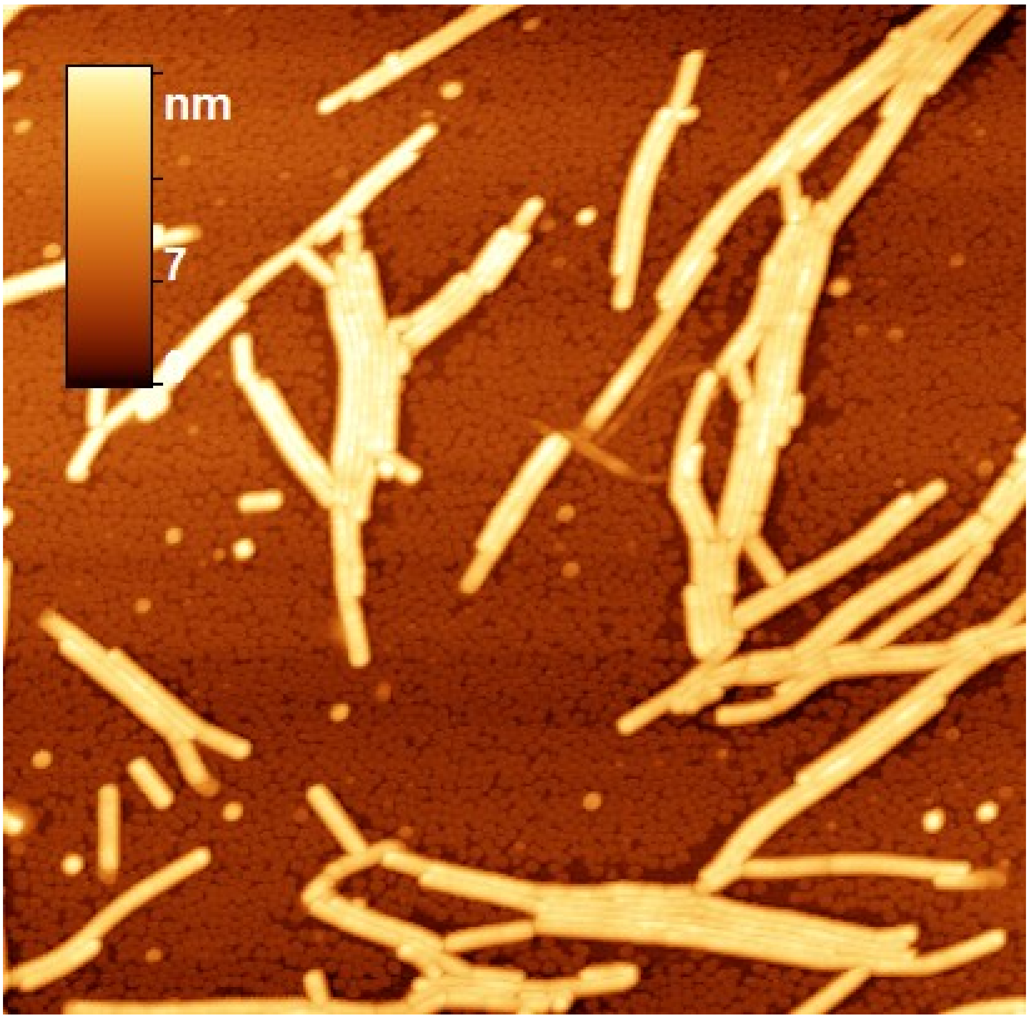

Figure S1. Atomic force microscopic analysis of TMV VLPs. The figure shows aggregates of rod-like particles with a height of 17.7 ± 0.5 nm, a diameter of 17.6 ± 2.0 nm, and a length of 330 ± 200 nm. The diameters were measured in clusters to avoid the probe broadening effect. A few disk-like assemblies of the coat protein lie on mica between the VLPs. Their height is 2.2 ± 0.3 nm.

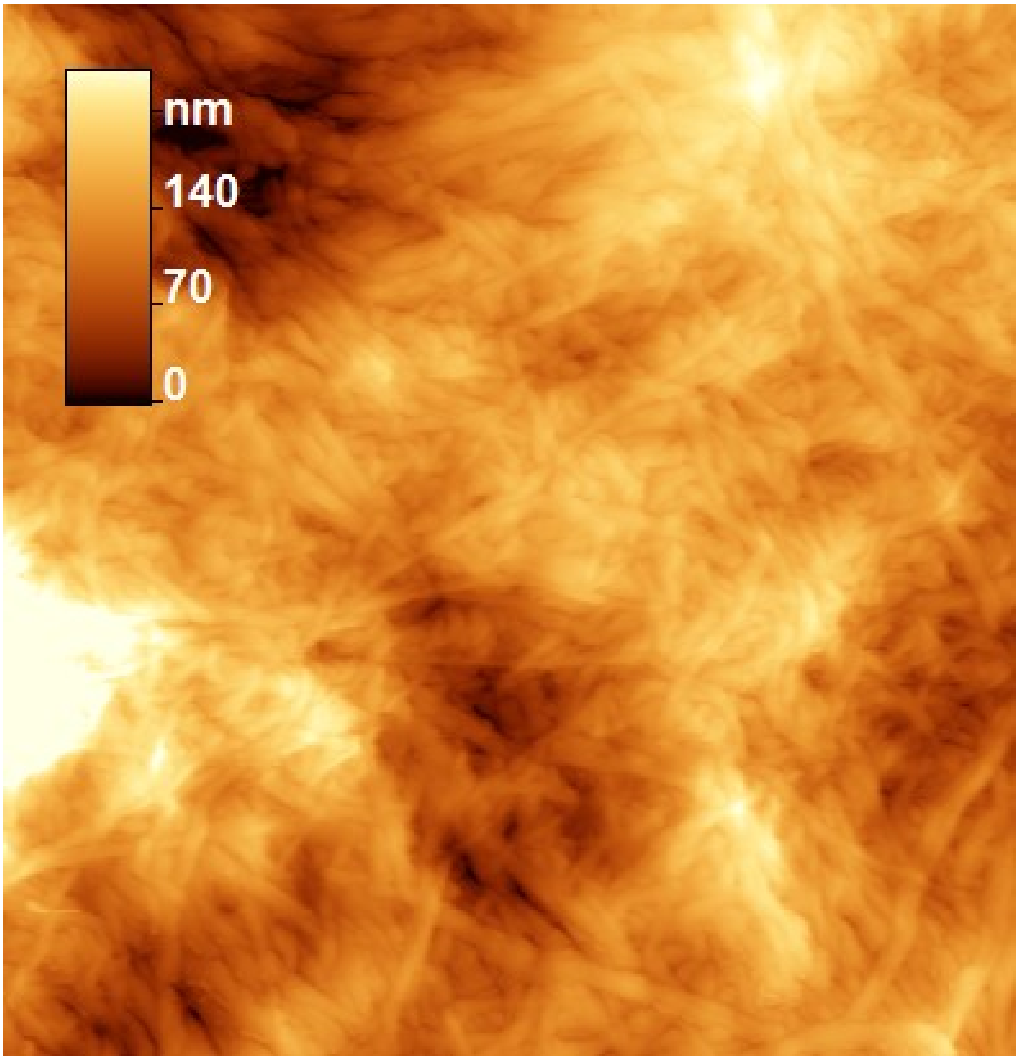

Figure S2. Atomic force microscopic analysis of nanocellulose. A nanocellulose film with random orientation of fibrils was found on the mica surface after sample drying. The diameter of the fibrils is 33 ± 7 nm.

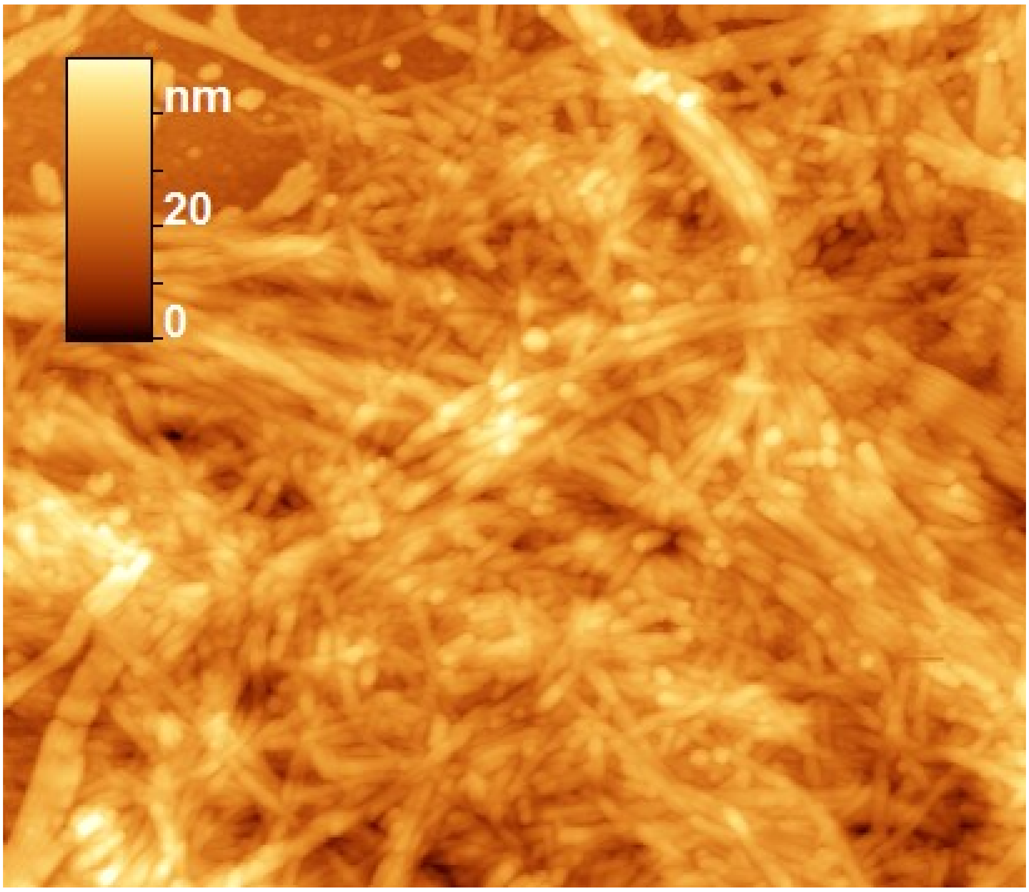

Figure S3. Atomic force microscopic analysis of TMV VLPs associated with the nanocellulose. The surface of the nanocellulose fibrils is mostly covered with aggregates of TMV VLPs.

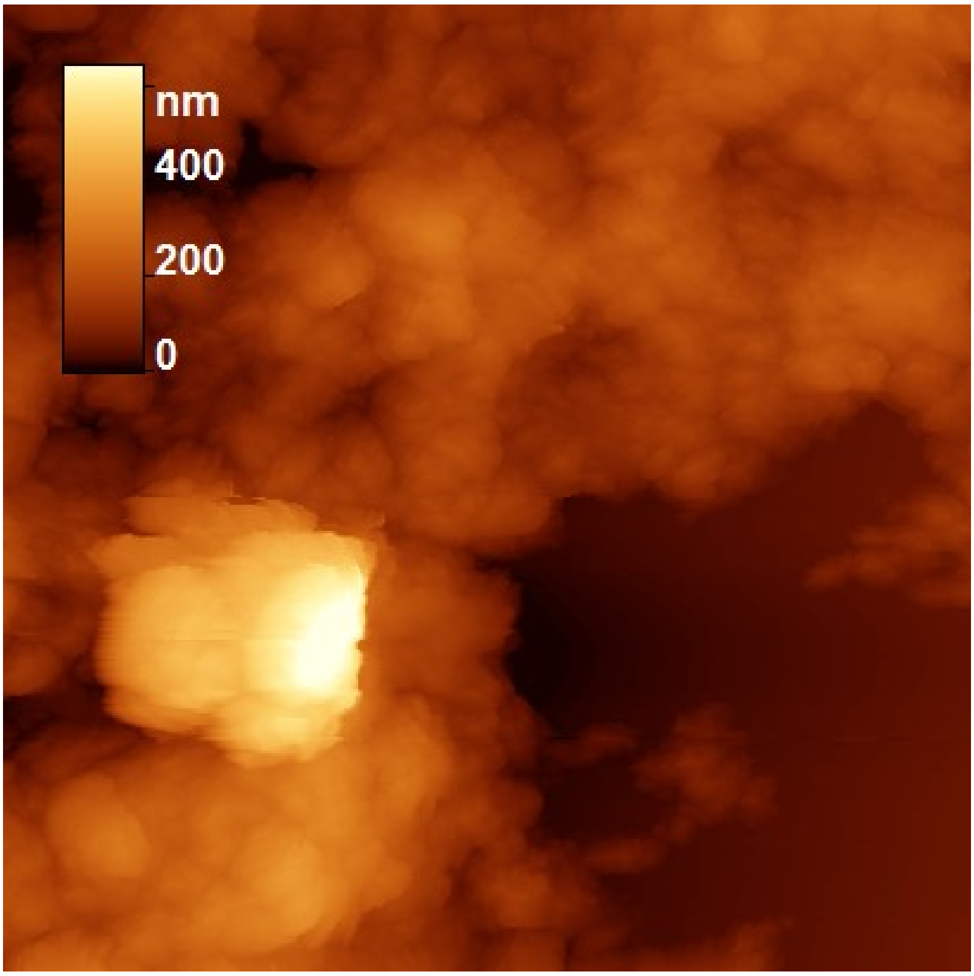

Figure S4. Atomic force microscopic analysis of TMV VLPs with nanocellulose after exposure to hydroxyapatite precursor salts (100 mM $CaCl_2$ pH 4.83 and 60 mM Na_2HPO_4 pH 8.36). Aggregates of plate-like crystals were found on the surface of mica after the sample drying.

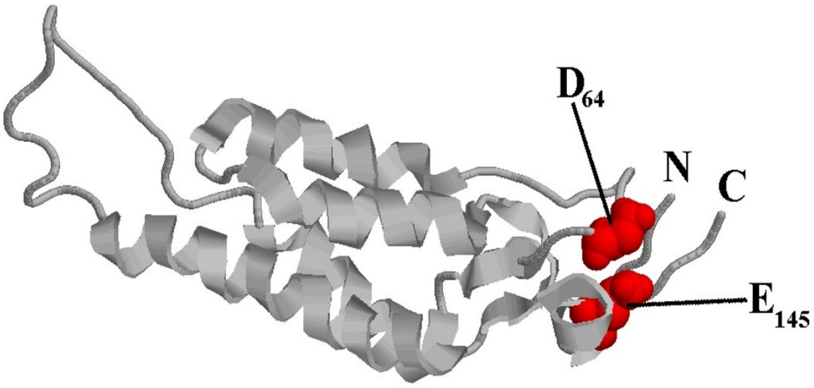

Figure S5. Ribbon structure of a TMV coat protein subunit, with N and C-terminals projecting to the virion outer surface, and the surface exposed Ca^{2+} sequestering amino acids highlighted.
